# The Impact of Social Media Use on Job Burnout: The Role of Social Comparison

**DOI:** 10.3389/fpubh.2020.588097

**Published:** 2020-11-19

**Authors:** Ruixia Han, Jian Xu, Yan Ge, Yulin Qin

**Affiliations:** ^1^School of Media and Communication, Shanghai Jiao Tong University, Shanghai, China; ^2^Institute of Cultural Innovation and Youth Development, Shanghai Jiao Tong University, Shanghai, China; ^3^China Institute for Urban Governance, Shanghai Jiao Tong University, Shanghai, China; ^4^Koguan School of Law, Shanghai Jiao Tong University, Shanghai, China

**Keywords:** social media, job burnout, social comparison, moderating role, mediating role

## Abstract

Through an online survey of a working population sample (*N* = 530), this study examines the role of social comparison between social media use and job burnout. The results show that: (1) there is a significant positive correlation between social media use and job burnout; (2) social comparison plays a moderating role in social media's impact on burnout. In high social comparative groups, the moderating role develops into an mediating role, which means that job burnout is only significant when social media addiction and the inclination of social comparison are simultaneously strong; (3) Social media users who often make downward comparison and get positive emotions from it are more prone to job burnout. This study reveals the possible negative effects of overuse of new media and enriches the understanding of how social media shapes individuals' psychology and behavior. Studies have also shown that regulating and controlling social comparisons and avoiding excessive use of social media may be effective in reducing job burnout.

## Introduction

Social media is fully involved in every aspect of our daily life. While it brings convenience to life, it also brings us a lot of trouble. For example, social media can provide professional information and network support, but it also consumes users' time and energy, which may interfere with life order and disrupt psychological balance.

Social psychology shows that people constantly judge their own living conditions by obtaining information from others. This kind of psychological activity, namely social comparison, is an important part of social cognition, by which individuals can realize their own social situation or state evaluation. Nowadays, social media has significantly improved the efficiency of obtaining information from others, so we could speculate that social media has the function of promoting social comparison. Social media may increase the frequency and intensity of individual's self-evaluation of appearance, health, family, wealth, and occupation. This study focuses on the impact of social media usage on personal professional status, especially on the relationship between social media (WeChat) usage and job burnout.

At the same time, we also found that in previous studies, people paid more attention to the relationship between different types of social comparisons, such as upward comparison, downward comparison, and job burnout. For example, Buunk et al. (1) found that compared with downward comparison, upward comparison is more likely to trigger positive emotions, less negative emotions, and people with more negative emotions have a higher level of job burnout. The findings of Carmona et al. (2) also show that downward identification and upward contrast are more likely to have a positive correlation with job burnout, while upward identification is negatively related to job burnout. These studies all indicate the possible impact of upward comparison and downward comparison on job burnout, especially the emotional effect caused by this comparison. Now that social media is widely used, has the relationship between different social comparisons and job burnout changed? In the social media space, what kind of associations are there between different types of social comparisons, the emotions they cause and job burnout?

## Literature Review and Research Hypotheses

### WeChat Addiction and Job Burnout

As early as 1969, Bradley proposed the notion of burnout, or job burnout (3), but it was Freudenberge (4) who first discussed it as a proper academic concept. The word “burn out” was first used to describe the outcome of chronic drug use. Freudenberge borrowed the term to describe the phenomenon in which hospital volunteers' enthusiasm, motivation, and commitment reduced after one year of work and they showed symptoms of psychological and physical disorder. Because such phenomena are common in the professional life of modern society, Freudenberge's research has attracted widespread attention (5). At present, researchers usually adopt Maslach's definition of the phenomenon from three dimensions. Job burnout is a long-term response to continuous emotional and interpersonal stress at work. It includes exhaustion, cynicism, and sense of inefficacy (6). Related research demonstrates that at the individual level, its influencing factors include role pressure, employee autonomy, work pressure, social support, high-intensity interpersonal interactions, job roles, and demographic variables, etc. At the environmental and organizational level, they include leadership style, reward and punishment methods, Job autonomy, decision-making opportunities, and values in the organization (7). The job demands-resources model (8) and the psychological capital model (9) analyze job burnout on both levels and are currently the most commonly used research models.

With the widespread use of social media, researchers have begun to focus on social media's relationship to professional feelings. For example, using the work-demand-resource model, Demerouti et al. (8) have found that social media can alleviate employees' work pressure. Nabi et al. (10) discover that social support by social media can reduce the emotional exhaustion caused by work. Moqbel et al. (11) demonstrates that social media can reduce the stress caused by work conflicts. Schouten et al. (12) shows that social media increases interaction between colleagues and reduces colleagues' alienation at work. Valkenburg et al.'s (13) research shows that Facebook has increased the sense of work participation and the sense of accomplishment of employees. In general, from the perspective of social support and social compensation theory, previous studies have emphasized that the use of social media can improve professional experience and reduce job burnout.

A relatively neglected but important phenomenon is the problem of overuse of social media, also known as social media addiction. Borrowing from cognitive-behavioral theory, social cognitive theory, and human-environment model, Zheng (14) shows that the excessive use of social media has a negative impact on family relationship, general interpersonal relationship, work, and study. Through a survey of Thai corporate employees, Sriwilai and Charoensukmongkol (15) observe that social media addiction reduces the use of problem-oriented work patterns and improves the use of emotionally-oriented work patterns, which triggers emotions related to burnout depletion. Research by Junco shows that the excessive use of social media at work can distract attention, affect job performance, and cause burnout. He also believes that social media usage reduces face-to-face interactions among colleagues, creates a sense of alienation, and promotes a sense of burnout (16). Valkenburg et al. (13) have shown that negative feedback received from social media can hurt personal accomplishment and cause burnout. In a review of relevant research, Andreassen (17) points out that social media addiction can have negative effects on multiple aspects of individual health, psychology, life, and work performance. Based on this, we propose the first hypothesis of this study:

H1: There is a positive correlation between social media usage and job burnout.

### Social Media Use and Social Comparison

Facebook has 2 billion users globally (18) and WeChat users have exceeded 1 billion (19). On social media, users present much personal information, including achievements, attitudes, activities, personalities, social relationships, and daily habits etc., to build their own image and provide a window for people to understand each other. There is reason to speculate that people have more opportunities to compare with others.

Related research supports the above speculation. Joinson (20) argues that more users spend time “diving” or viewing other people's Facebook activities than “posting” information about themselves. Vogel et al. (21) have shown that social media makes society more efficient and that those who are more prone to society will use Facebook more frequently, which also means that they are more likely to acquire negative feelings or low self-evaluation. Sagioglou and Greitemeyer (22) states that the longer individuals use Facebook, the stronger are their negative emotions. One of the reasons is that browsing other people's information leads to thinking about what they mean. Nesi and Prinstein (23) observes that social media usage is related to adolescents' depression and that frequent use and excessive search will strengthen adolescents' sense of social comparison, which may bring unhappiness. Xie and An (24) believe that social media's notion of sharing can easily induce users to evaluate their wealth, happiness, and success. Based on this, we propose the second hypothesis of this study:

H2: There is a positive correlation between social media usage and social comparison.

### Social Comparison and Job Burnout

Festinger (25) introduced the concept of social comparison in 1954. Generally speaking, social comparison refers to the psychological activities of individuals with others in terms of abilities, wealth, and social status. Social comparison helps individuals to establish Self-awareness. In Festinger's view, social comparison provides the internal psychological drive for individual's continuous pursuit of upward social mobility and thereby promotes the development of human society. According to Buunk and Gibbons (26), it is precisely through comparison that human beings confirm their own conditions, resist threats, and gain a sense of belonging. Social comparison includes upward social comparison, downward social comparison, and constructive social comparison (27). Different types of social comparison correspond to different social situations and have different social consequences.

Related research on burnout always takes social comparison as their concern. For Maslach et al. (28), feelings such as emotional exhaustion and reduced sense of accomplishment in job burnout are mostly derived from comparisons with colleagues in similar situations. Michinov (29) shows that socially-perceived control can effectively reduce job burnout. Kitchel et al. (30) find that social comparison plays a mediating role between job satisfaction and job burnout.

Chinese researchers have also noticed the relationship between social comparison and job burnout. For example, Zhao et al. (31) show that, in comparison to colleagues who get promoted, the higher the similarity is, and the larger the power gap is, knowledge-oriented employees are more likely to feel jealous and give up to job burnout. Jiang (32) discovers that both the upward comparison and downward identification create job burnout, whereas upward identification and downward comparison can effectively reduce burnout. Based on these findings, we propose a third hypothesis for this study:

H3: There is a positive correlation between social comparison and job burnout.

### Social Media Usage, Social Comparison and Job Burnout

**Figure 1:**
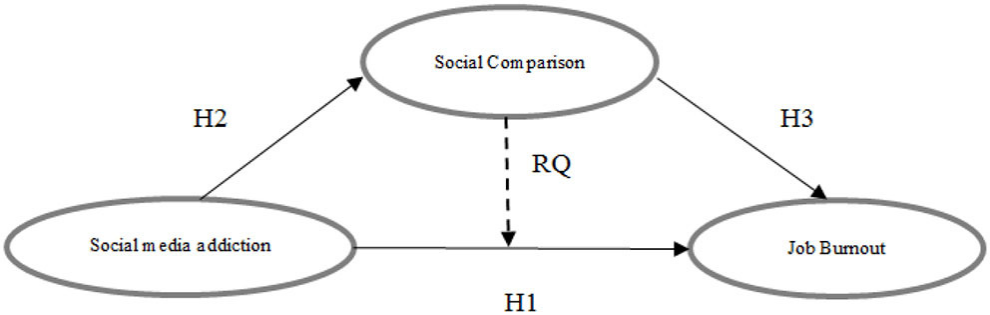
A proposed Core Model.

The above empirical research shows that social media usage is positively related to job burnout and social comparison, while social comparison is also positively related to job burnout. Logically, social media provides more possibilities for social comparison, and more social comparisons will trigger more frequent self-evaluation. Evaluation may be positive or negative, but burnout is a negative evaluation. The question is what is the more specific role of social comparison in connecting social media usage and job burnout? In other words, as a psychological mechanism, does social comparison play a moderating role, mediating role, or no obvious effect? Previous studies have provided the possibility of the three connections, but they have not explored the relationship or the mechanism of action in detail. So if social comparison has a moderating or moderating effect between social media addiction and job burnout, what is the conditional mechanism for this effect? Further question: What type of social comparison is more likely to play a role between social media addiction and job burnout? With these questions we conducted an exploratory study. The core issues and design of the research are as follows:

RQ: Does social comparison moderate or mediate social media's impact on job burnout? The research model is shown in Figure 1.

## Research Design and Implementation

### Sample and Data Collection

In Chinese social media, WeChat has many users. We use WeChat users as a sample group of social media and issue questionnaires through the online survey platform (https://www.wjx.cn/)(May 10-15, 2019). Using “on-the-job” and “whether to use WeChat” as sample screening questions, we randomly invited volunteers from a sample database of up to 2.6 million people to participate in the survey. In addition to screening questions, we also set IP addresses, computers, mobile phones, user restrictions, and logical questions to ensure that each sample can only be filled out once. A total of 530 valid questionnaires were eventually recovered (Table 1). Valid questionnaire recovery rate is 89%. *T*-tests of the first 25% and last 25% of the demographic data and all variables in the survey showed that there was no non-response bias at a confidence interval of 0.01.

**Table 1:** Distribution of sample socio-demographics.

	**Categories**	**Frequency**	**Percentage (%)**
Gender	Male	246	44.7
	Female	314	55.3
Education	Junior high school and below	6	1.1
	High school	15	1.7
	College/University	461	83.8
	Master	64	11.6
	Doctor and above	4	0.7
Income per year (Rmb)	<10,000	58	10.5
	10,000–30,000	49	8.9
	30,000–50,000	48	8.7
	50,000–100,000	186	33.8
	100,000–200,000	168	30.5
	200,000–500,000	34	6.2
	>500,000	7	1.3
Age	Mean	30.5	
Years of Current Job	Mean	6.61	

### Measurement

#### Burnout

We use the common Marl Burnout Inventory-General Survey (MBI-GS) to measure burnout. The Chinese version of the scale was revised by Li et al. (33). It contains 16 questions. Among them, 11-16 questions are reversed scoring, using Likert 7-point scoring (1 = never−7 = daily). The three dimensions of the question are emotional exhaustion, cynical (work alienation) and low self-fulfillment. Internal consistency (Cronbach's Alpha = 0.888), KMO and bartlett spherical test results (0.915) and significance (*p* = 0.000) meet the use standards.

#### Social Media Usage

For the measurement of social media usage, we adopt the widely employed the social media addiction scale compiled by Jamal Al-Menayes (34), only replacing “Social Media” with “WeChat.” The main questions are “I'm in a bad mood after being interrupted using WeChat,” “I like to cancel a meeting when I like to communicate on WeChat,” “I find that I have used WeChat for more than the original plan,” “Broken WeChat will make me feel insecure,” and “Ignored work or study because of WeChat,” etc., There are 10 questions in total. The scale uses Likert 5-point scoring (1 = never−5 = very frequently), internal consistency (Cronbach's Alpha = 0.735), KMO and bartlett spherical test results (0.776), significance (*p* = 0.000) meet the use standards.

#### Social Comparison

Social comparison is measured by the scale made by Gibbons and Buunk. Its Chinese version has been revised by Wan et al. (35). The questionnaire contains 11 questions. They include “I always pay close attention to the difference between how I do things with others,” “I often treat what my loved one (boy or girlfriend, family member, etc.) is doing and others,” “I often compare what I do with others in life,” and so on. Our design also includes two reverse scoring questions. The scale uses Likert 5-point scoring (1 = never−5 = very frequently). Internal consistency (Cronbach's Alpha = 0.832), KMO and bartlett spherical test results (0.881), significance (*p* = 0.000) all meet the use criteria.

#### Demographic Variables

Existing research shows that gender, age, and current working hours may affect job burnout, so we put it in the regression model as a background variable. The test finds that the type of occupation and education do not significantly affect job burnout. In order to maintain the consistency of the measurement variable levels, we exclude them from the overall model.

### Statistical Analysis

We adopted hierarchical regression method by spss19.0 to test relevant research hypotheses. In the test of mediation or moderation, we mainly adopt causal step regression method of Baron and Kenny (36). However, as pointed out by subsequent researchers such as Hayes (37), causal step regression cannot provide relevant data such as confidence intervals and effect sizes.

## Findings

### Correlation Test

Correlation tests show that WeChat usage has a significant and positive correlation with both job burnout and social comparison. There is no significant correlation between social comparison and job burnout. In other words, samples using WeChat more frequently have a stronger sense of job burnout and a stronger tendency for social comparison (see bold values in Table 2). However, samples more subject to social comparison do not necessarily feel job burnout. In addition, older people are more prone to burnout (Table 2).

**Table 2:** Result of correlation test (*N* = 530).

**Variables**	**Age**	**Years of Current Job**	**Wechat Use**	**Social Comparison**	**Job Burnout**
**Age**					
Years of Current Job	0.121**				
Wechat Use	−0.075	−0.037			
Social Comparison	0.080	0.081	0.317**		
Job Burnout	0.121**	0.068	0.119**	0.020	
Mean	10.72	6.61	2.9736	2.8300	4.9875
Sd	5.857	5.080	0.59230	0.67837	0.85416

**p < 0.01.

### Regression Analysis and Hypothesis Testing

We conduct a regression analysis to test our research hypotheses (Table 3). Model3-1 uses demographic variables as independent variables and finds that the age variables have a significant effect on job burnout. Model3-2 introduces the use of independent variable WeChat and shows that WeChat usage has a significant and positive impact on burnout, which supports H1. Model 3 introduces social comparison and discovers that social comparison has no significant effect on job burnout, which denies H3. Model3-4 introduces two variables, WeChat usage and social comparison, and finds that WeChat usage has a significant impact on job burnout but social comparison has no significant impact on job burnout. Model3-5 introduces as its variable the interacting effect of WeChat usage and social comparison, and finds that the significant effect of WeChat usage in Model3-2 and Model3-4 no longer exists, but social comparison and its interacting effect with WeChat usage are significant. This indicates that social comparison moderates between WeChat usage and job burnout. In addition, Model3-5 also gets the highest adjusted *R*^2^. This finding partially answers RQ.

**Table 3:** Multiple Regression Analysis for Job Burnout (Standard coefficient; *N* = 530).

		**M3-1**	**M 3-2**	**M 3-3**	**M 3-4**	**M 3-5**
	Gender	0.054	0.067	0.053	0.074	0.074
	Age	0.178*	0.194**	0.178**	0.198**	0.208**
	Years of Current Job	−0.066	−0.072	−0.066	−0.070	−0.077
Independent variable	Wechat Use		0.137**		0.153**	−0.227
Moderating variable	Social Comparison			0.005	−0.047	−0.508*
Interaction variable	Wechat Use × Social Comparison					0.691*
	*F*	3.500*	5.178***	2.623*	4.351**	4.553***
	Adjusted *R*^2^	0.014	0.031	0.012	0.031	0.139

*p < 0.05, **p < 0.01, ***p < 0.001.

In order to better understand the role of social comparison, we also conducted a regression analysis on the data of the high social comparison tendency sample (the top 25% of the score) (Table 4). Model4-2 shows that WeChat usage has a significant effect on burnout. Model4-3 shows that social comparison also has a significant effect on burnout. Model4-4 shows that the introduction of WeChat usage has no significant effect on job burnout. Model4-5 further shows that WeChat usage, social comparison, and their interaction have significant effects on job burnout. This shows that, in the sample of high social comparison tendency, social comparison has a powerful effect beyond the use of WeChat. Conversely, it indicates that only when social comparison tendencies are more obvious and social media use is strong, job burnout is more likely to occur.

**Table 4:** Multi-level regression analysis model of job burnout in high social comparative groups (Standard coefficient; *N* = 133).

		**M4-1**	**M4-2**	**M4-3**	**M 4-4**	**M4-5**
	Gender	0.198*	0.231**	0.156	0.190*	0.200*
	Age	−0.004	0.082	−0.020	0.045	0.024
	Years of Current Job	0.210	0.146	0.234*	0.183	0.190
Independent variable	Wechat Use		0.251**		0.179	−2.380*
Moderating variable	Social Comparison			0.260**	0.198*	−1.029*
Interaction variable	Wechat Use × Social Comparison					3.155*
	*F*	3.181*	4.648**	4.961**	4.835***	5.205***
	Adjusted *R*^2^	0.047	0.100	0.107	0.127	0.160

*p < 0.05, **p < 0.01, ***p < 0.001.

### Simple Effects Analysis

**Figure 2:**
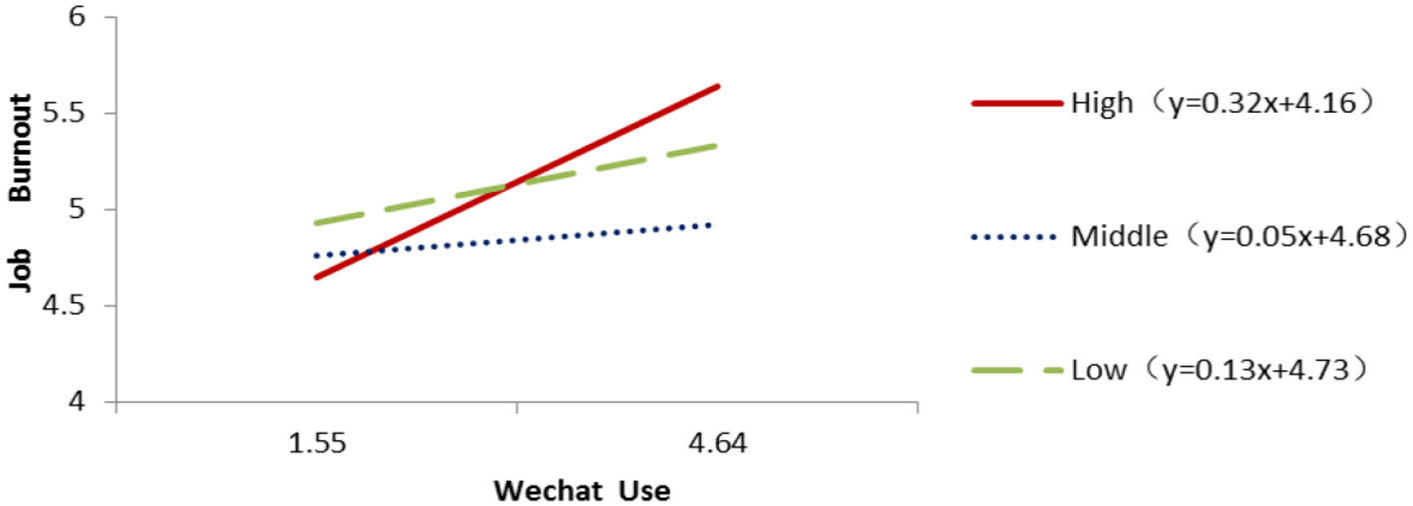
The relationship between Wechat Use and Job burnout under different social comparison levels.

The sample group is divided into high (25% before the score, *N* = 133), low (25% after the score, *N* = 133) and medium (score between the high and low, 50%, *N* = 264) according to the social tendency. Regression fit analysis of three groups shows that in the high propensity group, WeChat usage is significantly predictive of burnout (β = 0.32, *P* = 0.003, *R*^2^ = 0.064), whereas in the other two groups, the predictive power is not significant (medium: β = 0.05, *P* = 0.58, *R*^2^ = 0.001; Low: β = 0.13, *P* = 0.34, *R*^2^ = 0.007). Figure 2 shows plainly that when the sample society is more inclined and indulged in WeChat, job burnout is more likely to occur.

### Extended Analysis: WeChat Users' Upward and Downward Comparison and Its Impact on Job Burnout

In order to further explore how social comparison affects the job burnout of WeChat users, we introduce two categories of social comparison: upward comparison and downward comparison. We also introduce into our regression analysis four independent variables, which include the positive emotions caused by upward comparison, the negative emotions caused by upward comparison, the positive emotions caused by downward comparison, and the negative emotions caused by downward comparison. At the same time, the above four variables and the cross-variables formed by the use of WeChat are also introduced into the regression model to examine their impact on burnout. Table 5 displays the result of our multi-level regression analysis.

**Table 5:** Impact of upward comparison and downward comparison of emotion on WeChat Users' Burnout (Standard coefficient; *N* = 530).

		**M 5-1**	**M 5-2**	**M 5-3**	**M 5-4**
	Gender	0.075	0.075	0.067	0.067
	Age	0.010**	0.010**	0.009**	0.009**
	Years of Current Job	0.012	0.012	0.011	0.011
Independent variable	Wechat Use(WU)		0.062**	0.057*	0.400
Moderating variable	Pe by Upc			0.035***	0.181
	Ne by Upc			0.044**	0.238
	Pe by Dnc			0.034*	0.162**
	Ne by Dnc			0.037*	0.198
Interaction variable	Wu × Pe by Upc				0.059
	Wu × Ne by Upc				0.080
	Wu × Pe by Dnc				0.053**
	Wu × Ne by Dnc				0.067
	*F*	3.500*	5.178**	19.868***	14.681***
	Adjusted *R*^2^	0.014	0.031	0.222	0.237

*p < 0.05, **p < 0.01, ***p < 0.001.

In this study, we use the following four questions to measure the positive and negative emotions caused by upward and downward comparison. They are (1) finding colleagues or friends perform better in work and life than yourself makes you feel happy; (2) finding colleagues or friends perform better in work and life than yourself makes you feel unhappy; (3) finding colleagues or friends perform worse in work and life than yourself makes you feel happy; (4) finding colleagues or friends perform worse in work and life than yourself makes you feel unhappy. We use the four variable names of pe by upc, ne by upc, pe by dnc, and ne by dnc to represent them. Our simple correlation analysis indicates that they are correlated with WeChat addiction and burnout.

So how exactly social comparison plays its mediating role between WeChat usage and burnout? From the comparison of each model in Table 5, we see that when demographic variables and WeChat usage are considered separately, the overall explanatory power of models 5-1 and 5-2 is not high. When introducing pe by upc, ne by upc, pe by dnc, and ne by dnc, the four variables are significantly correlated with job burnout. The adjusted *R*^2^ of the entire model 5-3 reaches 0.222. When cross variables between the four variables and WeChat usage are also included in model 5-4, it can be found that the positive emotions caused by downward comparison (pe by dnc) and the cross variables formed with WeChat usage (wu × pe by dnc) have a more significant impact on burnout. The whole model adjusted *R*^2^ to reach 0.237. The results indicate that those WeChat users who prefer to compare themselves to those who are not as good as themselves are more likely to experience job burnout.

## Conclusion and Discussion

### Social Media Addiction Has a Significant Negative Impact on Burnout

The results show that there is a significant positive correlation between WeChat addiction and burnout. That is to say, those who are prone to WeChat addiction or WeChat in-depth users are more likely to have burnout. This confirms that in the age of social media, media has a deep impact on people's daily lives and enriches our life and work. Previous studies mostly focus on how addiction to WeChat affects adolescents' academic performance and psychology. However, they pay little attention to social media's impact on adults and their work. The limited amount of attention to adults concentrates on seeing social media merely as a positive tool to acquire “information” and “network connections.” However, this tends to ignore social media as a new platform for human life and its impact on the internal psychological mechanism of individuals. This study shows that social media can lead to job burnout. For the Chinese, there is a significant positive correlation between WeChat addiction and their job burnout. Understanding this fact is a prerequisite for understanding human psychological evolution in the era of social media and for intervening in social media's negative impact.

### There Is a Significant Positive Correlation Between Social Media Addiction and Social Comparison, but Social Comparison Alone Does Not Cause Job Burnout

Judging by the significant positive correlation between WeChat addiction and social comparison, correlation does exist between social media addiction and social comparison, regardless of whether WeChat addiction aggravates people's tendency for social comparison or people with a higher tendency for social comparison are more likely to become addicted to WeChat. This confirms the hypothesis raised by previous studies that defines social media as a large experimental field for social comparison. All kinds of information released by social media, directly or indirectly, provide the objects for people to do social comparison. Social comparison is bourn spontaneously from the human needs to adapt to the environment. This is proven by the lack of direct correlation between social comparison and job burnout, because the tendency for social comparison do not naturally cause burnout. On the contrary, the negative correlation coefficient in the comprehensive model 5 in Table 3 reveals that the tendency for social comparison tends to negatively affect job burnout. Those who have a higher tendency for social comparison usually do not have job burnout. Negative effects occur only when this psychological tendency is intersected with social media addiction. This demonstrates social media's significance in the era of media as the social space for social comparison.

### Social Comparison Plays a Moderating Role Between Social Media Addiction and Burnout

The regression model of job burnout in Table 3 shows that, with regard to the interactive relationship between social media (WeChat) addiction, social comparison, and job burnout, the influence of WeChat addiction is no longer significant, whereas social comparison and the interaction between social comparison and WeChat addiction become significant influencing factors. It fully illustrates the fact that WeChat addiction impacts job burnout through social comparison. This directly shows that social comparison critically mediates between social media addiction and job burnout. This is a further revelation of the role of social comparison in the age of social media. Most existing studies have noticed the impact of social comparison in real space on job burnout, but they did not notice the impact of the expansion of social comparative space in the age of social media on professional life. In terms of social media usage, people only pay attention to the use of social media to consume time and energy, reduce face-to-face interactions, and receive negative feedback and other physical meanings or direct psychological effects. In this role, this study effectively grafts the influence of individual psychological tendencies on individual's work and life in the era of social media. This study identifies the important role of social comparison in revealing the social and psychological logic of the “spectacle society” brought by social media.

### The Positive and Negative Emotions Triggered by the Upward and Downward Comparisons Have a Significant Impact on the Job Burnout of WeChat Users

The research results in Table 5 further explain how social comparison impacts job burnout among WeChat users. From this we can find that the positive and negative emotions caused by the upward and downward comparisons have a general impact on people's burnout, but when we consider the extent of WeChat usage, we find that those who often compare themselves to people that are inferior in living and working conditions in order to gain positive implications are more likely to feel job burnout. This research finding enriches our understanding of the relationship between social comparison and job burnout in the age of social media. It further expands the scope of the research by Buunk et al. (1) and Carmona et al. (2).

### The Role of Demographic Variables in the Relationship Between Social Media Addiction and Burnout

The results show that age and burnout are significantly and positively correlated. Older people have a stronger sense of burnout, which is demonstrated by Table 3, comprehensive model 5. For higher social comparison groups, women are more prone to have burnout. For the general social group, women have a stronger tendency for social comparison, whereas men are more likely to indulge themselves in WeChat. Relatively speaking, our research discovers that the effects of academic qualification and types of occupation on people's job burnout are not significant. This shows that there is no significant difference in the distribution of job burnout among different academic qualifications and different occupations.

## Implications and Limitation

This study demonstrates the important role that social comparison plays between social media addiction and burnout. Although there is a significant correlation between social media addiction and job burnout, this correlation works through social comparison. It reveals the logic that connects social media addiction and burnout and enriches people's understanding of the role of individual psychology in the social media era. It also shows that social media has indeed expanded the horizon of people's social comparison and that people have also adjusted their relationship with society through social comparison in the “media spectacle” era. They maintain a sense of security and belonging, resist social threats, and thereby gain social adaptation and integration, even though this result sometimes presents its negative appearance. From a practical perspective, this study tells us that to break the negative correlation between social media addiction and job burnout, we need to control the tendency for social comparison. Existing research has shown that social comparison is a process of thinking construction (29), which is similar to emotional experience. Individuals' recognition of their “social comparison” emotions and their understanding of the “spectacle” of social media information will effectively regulate the relationship between individual social media usage and burnout. Overall speaking, in the age of social media, social comparison remains to be a positive psychological tendency.

This study reveals the moderating role of social comparison in the relationship between social media usage and job burnout. However, whether there are other psychological tendencies (e.g., psychopathology) that will affect the relationship between the two is worth exploring and testing in future research. In terms of research methods, this study uses cross-sectional data. Since the time sequence of occurrence of related factors cannot be determined, further research is needed to determine the causality. At the same time, there are many types of social comparison. This study only analyses WeChat users' job burnout that is mediated by the positive and negative emotions caused by the upward and downward comparison. More detailed and rigorous analyses need further development. In addition, this study only focuses on WeChat, which is currently the most widely used social media by the Chinese. With regard to other social media such as Weibo, Tiktok, Kuaishou, Facebook and Line, it remains to be asked what the relationship between different media, different types of social media usage, and workplace mentality is. All of these studies will enrich our understanding of the psychological and behavioral changes in the age of social media.

Evolutionary psychology believes that social cognition is shaped by the environment. In order to adapt and control the environment, human beings have created many media for transmitting and receiving information, such as language, text, pen, and paper, radio, television, movies, and the Internet. Different media have different physical properties. These properties restrict and develop the cognitive mechanism that people use to obtain information and understand the world through media. There is reason to believe that as the latest information dissemination medium that is ubiquitous in life, social media inevitably adjust, change, and even reshape our cognitive style (38). Social media arises from our needs to adapt to the environment, but it has undoubtedly become an important part of the environment we need to adapt to. To be sure, social media brings us more information about the world. Questions need to be asked: will the use of social media for information acquisition and dissemination have a negative impact on reducing our ability to adapt to the environment? In what aspects do reductions happen? In what ways can it be improved? By explaining the relationship between WeChat usage and burnout, this study attempts to contribute to answering these questions.

## Data Availability Statement

The datasets generated for this study can be found in online repositories. The names of the repository/repositories and accession number(s) can be found below: annahan08@sjtu.edu.cn.

## Author Contributions

RH and JX: conceptualization, funding acquisition, and investigation. RH and YG: methodology. RH and YQ: data curation and formal analysis. JX: supervision. RH: writing–original draft. RH, YG, and JX: writing–review and editing. All authors have read and agreed to the published version of the manuscript.

## Conflict of Interest

The authors declare that the research was conducted in the absence of any commercial or financial relationships that could be construed as a potential conflict of interest.
